# Evaluation of right ventricular systolic and diastolic dysfunctions in patients with type 2 diabetes mellitus with poor glycemic control by layer specific global longitudinal strain and strain rate

**DOI:** 10.1186/s13098-022-00820-1

**Published:** 2022-04-08

**Authors:** Jun Huang, Li Li, Li Fan, Dong-liang Chen

**Affiliations:** 1 Department of Echocardiography, the Affiliated Changzhou No.2 People’s Hospital With Nanjing Medical University, Changzhou, 213003 China; 2 Department of Pediatrics, Changzhou Fourth People′S Hospital, Changzhou Tumor Hospital Affiliated to Soochow University, Changzhou, 213003 China

**Keywords:** Type 2 diabetes mellitus, Right ventricle, Systolic, Diastole, Dysfunction

## Abstract

**Background:**

In order to evaluate right ventricular (RV) systolic and diastolic dysfunctions in patients with type 2 diabetes mellitus (T2DM) with poor glycemic control by layer specific global longitudinal strain (GLS) and strain rate (GLSr).

**Methods:**

68 T2DM patients and 66 normal controls were enrolled for the present study. RV layer specific GLS (GLSEpi, GLSMid and GLSEndo represent the epimyocardial, middle layer and endomyocardial strains, respectively) and GLSr (GLSr-S, GLSr-E and GLSr-A represent the systole, early-diastole and late-diastole strain rate) were calculated by averaging each of the three regional peak systolic strains and strain rates along the entire RV free-wall (RVFW), entire RV free-wall and septal wall (RVFSW) on RV-focused view.

**Results:**

The absolute values of RV layer specific GLS (GLSEpi, GLSMid and GLSEndo) in RVFW in T2DM patients were significantly lower than normal controls (*P* < 0.01), while GLSr-A was significantly larger than normal controls (*P* < 0.001). The absolute values of RV layer specific GLS (GLSEpi and GLSMid) in RVFSW in T2DM patients were significantly lower than normal controls (*P* < 0.05), while GLSr-A was significantly larger than normal controls (*P* < 0.001). HbA1c were poor negatived correlated with GLSEpi in RVFW and RVFSW in T2DM patients (*P* < 0.05). ROC analysis showed that RV layer specific GLS and GLSr-A had a high diagnostic efficacy in T2DM patients, and GLSr-A in RVFSW have the best diagnostic value in RV diastolic function in T2DM patients (AUC: 0.773).

**Conclusion:**

From the research, we found that layer specific GLS and GLSr could detect the RV myocardial dysfunctions and confirmed that the impaired RV systolic and diastole functions in T2DM patients with poor glycemic control. GLSr-A in RVFSW had the best diagnostic value in evaluating RV diastolic function in T2DM patients.

## Background

The prevalence of type 2 diabetes mellitus (T2DM) is increasing in recent years. T2DM is characterized by a low-grade inflammatory status and endothelial dysfunction, which substantially potentiates the risk of developing cardiovascular diseases [1]. T2DM can damage the myocardial by itself, in patients without any evidence of coronary artery disease, hypertension, and valvular heart disease [2]. Diabetes-related cardiomyopathy, known as diabetic cardiomyopathy is paid more and more attention by the endocrinologists and cardiologists, possibly leads to heart failure with preserved left ventricle ejection fraction (LVEF) [3]. However, many researches were focused on the left ventricular (LV) subclinical myocardial dysfunctions in T2DM patients because they considered that LV plays a key role for maintaining the cardiac functions [4–8]. As the lower chamber of the cardiac and the complex geometry, non-uniform contraction and partly retrosternal position of the right ventricle (RV) [9], the assessment of RV function remains little and difficult in T2DM patients.

At present, the main techniques to evaluate RV function in T2DM patients mainly contain echocardiography and cardiac magnetic resonance (CMR). Hu BY, et al. [10] used CMR feature tracking (CMR-FT) to determine the RV function in T2DM patients, and found in T2DM patients, CMR-FT could quantify RV deformation and identify subclinical RV dysfunction in those with normal RVEF. Tadic M, et al. [11] found that T2DM and hypertension significantly influence RV mechanics assessed by two-dimensional echocardiography (2DE) conventional and 2DE multilayer strain. Speckle tracking imaging (STI) based on the echocardiographic is considered as the a convenient, flexible and accurate method for evaluating RV function in different cardiac disorders.

The aim of the investigation was to determine layer specific global longitudinal strain (GLS) and strain rate (GLSr) of RV myocardium in T2DM patients with poor glycemic control and without any cardiovascular diseases. Furthermore, we attempted to evaluate the association between laboratory parameters of T2DM patients, echocardiographic characteristic and indices of RV structural, functional and mechanical remodeling in the study population.

## Subjects and methods

### Ethical statement

The present study was approved by the Human Research and Ethics Committee of the Affiliated Changzhou No. 2 People’s Hospital with Nanjing Medical University. All patients completed the informed consent forms.

### Study population

Our study included 68 untreated T2DM patients (not well-treated, and it means poor blood glucose control in these T2DM patients before hospitalization) and 66 normal controls of similar age and gender. The criteria for T2DM patients were clinically confirmed in accordance with the World Health Organization criteria [12], subjects with history of heart disease (congenital heart disease, coronary artery disease, arterial hypertension, myocardial infarction, cardiomyopathy, valvular disease, atrial fibrillation, thyroid disease, neoplastic disease, or kidney failure), obesity and dyslipidemia were excluded from the study. All enrolled subjects were performed with coronary CT or coronary angiography to confirm that they have no coronary artery disease.

### Anthropometric and biochemistry

Anthropometric measures, such as height, weight, heart rate, blood pressure (systolic blood pressure: SBP, diastolic blood pressure: DBP) and biochemistry analyses, such as fasting plasma glucose, glycated haemoglobin (HbA1c), total cholesterol (TCH), triglyceride (TG), high density lipoprotein (HDL) and low density lipoprotein (LDL) were taken from all the subjects included in the study when the patients were in hospital.

### Conventional 2D Doppler echocardiography

Patients underwent conventional 2D transthoracic Doppler echocardiography with a Vivid E9 equipped with an M5S 3.5–5 MHz transducer (GE Vingmed Ultrasound, Horten, Norway) by an experienced cardiologist. ECG was recorded synchronously at rest. RV-focused view of three consecutive cycles with a standard high frame rate (> 45 s^−1^) were stored for offline analysis. The RV middle diameter and RV basal diameter of the T2DM group and normal control group were measured in the RV-focused view. The RV areas in the diastole and systole period were measured, and then the RV fractional area change (RVFAC) was calculated. RV end diastole and systole volume were measured and the RV ejection fraction (RVEF) were calculated. LVEF was calculated by the bi-plane Simpson′s method. The tricuspid annular plane systolic excursion (TAPSE) was measured in M-mode. The early diastolic velocity (E) and late diastolic velocity (A) of the tricuspid valve were measured by pulsed wave Doppler. The early diastolic (e′) and late diastolic velocities (a′) of the anterior tricuspid annulus were measured by tissue Doppler. Tricuspid regurgitation velocities were assessed by continuous wave color Doppler in RV-focused view.

### Two-dimensional speckle tracking echocardiography

**Fig. 1:**
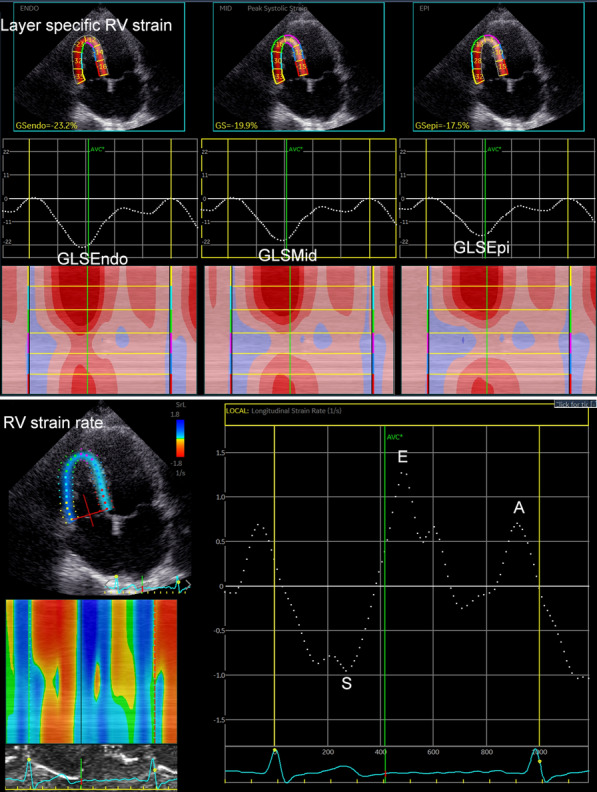
The measurement of layer specific global RV longitudinal strain, strain rate in normal controls and T2DM patients

RV layer specific GLS and GLSr were performed by EchoPAC software (EchoPAC Version: 203, GE Vingmed Ultrasound, Norway) in RV-focused view by averaging all the values in the regional peak longitudinal strain and strain rate. RV layer specific free-wall strain (GLSEpi, GLSMid and GLSEndo represented the epimyocardial, middle layer and endomyocardial strains, respectively) and strain rate (GLSr-S, GLSr-E and GLSr-A represent the systole, early-diastole and late-diastole strain rate) were calculated by averaging each of the three regional peak systolic strains and strain rates along the entire RV free-wall (RVFW), while RV free and septal wall strain (GLSEpi, GLSMid and GLSEndo represent the epimyocardial, middle layer and endomyocardial strains, respectively) and strain rate (GLSr-S, GLSr-E and GLSr-A represent the systole, early-diastole and late-diastole strain rate) were calculated by averaging each of the six regional peak systolic strains and strain rates along the entire RV free-wall and septal wall (RVFSW). RV systolic functions contain: GLSEpi, GLSMid, GLSEndo, GLSr-S in RVFW and in RVFSW, and RV diastole functions contain: GLSr-E, GLSr-A in RVFW and in RVFSW (Fig. 1).

### Statistical analysis

All data analyses were performed using SPSS 25.0 software (SPSS, Chicago, IL, USA). The Kolmogorov–Smirnov test was used to detect the normality of all values. Continuous variables are expressed as mean values with standard deviation for normally distributed data and median values with interquartile range for non-normally distributed data. Categorical variables are expressed as frequencies and percentages. Differences between the T2DM patients and normal controls were compared with an independent Student’s *t*-test or Mann–Whitney test as appropriate. Chi square test was used to compare the rates. Correlations among biochemistry, echocardiography characters and layer-specific RV GLS and GLSr values were tested using Pearson or Spearman correlation tests as appropriate. Receiver operating characteristic (ROC) curve analysis were performed by MedCalc software to confirm the diagnostic values of GLS and GLSr. Youden’s index was selected as the cut-off point that can give the best composite of specificity and sensitivity. A *P*-value < 0.05 was considered significant in all tests.

## Results

### Patient characteristics

A total of 134 patients met the baseline inclusion criteria. 23 patients were excluded from the strain and MW analyses because of inadequate image quality (n = 9), tachycardia (n = 6) or irregular heartbeat (n = 8). A total of 111 patients were therefore evaluated in the study and were initially divided into two groups: normal controls (n = 59, mean age: 50.51 ± 13.14 years, men: 33) and T2DM patients (n = 52, mean age: 54.54 ± 11.42 years, men: 30).

### Clinical characteristics and conventional echocardiographic data were shown in Table1

**Table 1:** Baseline clinical characteristics, conventional two-dimensional echocardiographic parameters between T2DM patients and normal subjects (mean ± SD)

Variable	Normal (59)	T2DM (52)	*t/χ2* value	*P* value
Clinical				
Age (years)	50.51 ± 13.14	54.54 ± 11.42	− 1.728	0.087
Male (%)	33(59)	30(62)	0.035	0.852
BMI (kg/m^2^)	23.23 ± 3.13	23.89 ± 3.08	− 1.132	0.260
Heart rate (bpm)	75.12 ± 9.18	76.21 ± 9.61	− 0.611	0.543
DBP (mmHg)	123 ± 12	127 ± 13	− 2.053	0.043
SBP (mmHg)	76 ± 9	78 ± 9	− 0.938	0.351
Fasting plasma glucose (mmol/L)	4.88 ± 0.62	13.27 ± 4.23	− 15.055	< 0.001
HbA1c (%)	5.09 ± 0.61	10.20 ± 2.14	− 17.564	< 0.001
TCH (mmol/L)	3.88 ± 0.69	3.97 ± 0.80	− 0.680	0.498
TG (mmol/L)	1.11 ± 0.30	1.21 ± 0.59	− 1.168	0.245
HDL (mmol/L)	1.25 ± 0.28	1.19 ± 0.34	1.053	0.295
LDL (mmol/L)	2.04 ± 0.49	2.21 ± 0.59	− 1.662	0.100
Medical treatment				
Diet treatment	0(0)	0(0)		
Oral drug	0(0)	16(31)		
Insulin	0(0)	32(62)		
Insulin + oral drug	0(0)	15(29)		
Echocardiography				
RVd-base (mm)	30.25 ± 4.09	30.31 ± 3.09	− 0.092	0.927
RVd-mid (mm)	25.90 ± 3.85	25.73 ± 4.74	0.211	0.834
RVA-D (cm^2^)	14.21 ± 2.75	13.58 ± 2.73	1.217	0.226
RVA-S (cm^2^)	6.88 ± 1.82	6.56 ± 1.73	0.935	0.352
RVFAC (%)	51.82 ± 6.67	51.90 ± 6.92	− 0.067	0.946
TAPSE (mm)	22.92 ± 3.16	23.04 ± 2.54	− 0.225	0.823
RVEDV (ml)	26.10 ± 8.83	24.73 ± 9.12	0.802	0.425
RVESV (ml)	9.07 ± 3.49	8.50 ± 3.48	0.856	0.394
RVEF (%)	65.51 ± 5.62	65.63 ± 6.65	− 0.107	0.915
LVEF (%)	64.78 ± 4.56	62.35 ± 4.87	2.738	0.007
E (m/s)	0.58 ± 0.10	0.54 ± 0.09	2.448	0.016
A (m/s)	0.37 ± 0.08	0.37 ± 0.07	0.418	0.677
E/A	1.60 ± 0.31	1.48 ± 0.24	2.183	0.031
e′(m/s)	0.15 ± 0.03	0.13 ± 0.04	2.532	0.013
a′ (m/s)	0.14 ± 0.04	0.12 ± 0.02	1.999	0.048
E/e′	4.08 ± 0.80	4.55 ± 1.95	− 1.711	0.090
TRV (m/s)	2.05 ± 0.37	2.13 ± 0.30	− 1.369	0.174

*BMI* body mass index, *SBP* systolic blood pressure, *DBP* diastolic blood pressure, *HbA1c* glycated haemoglobin, *TCH* total cholesterol, *TG* triglyceride, *HDL* high density lipoprotein, *LDL* low density lipoprotein, *RVd-base* right ventricle basal diameter, *RVd-mid* RV middle diameter, *RVA-D* RV areas in the diastole period, *RVA-S* RV areas in the systole period, *RVFAC* RV fractional area change, *RVEDV* RV end diastole volume, *RVESV* RV end systole volume, *RVEF* right ventricular ejection fraction, *LVEF* left ventricular ejection fraction, *E* peak velocity during early diastole of tricuspid valve, *A* peak velocity during late diastole of tricuspid valve, *e′* peak early diastolic velocities of the anterior tricuspid annulus using TDI, *a*′ peak late diastolic velocities of the anterior tricuspid annulus using TDI, *TRV* tricuspid regurgitation velocity

The values of SBP, fasting plasma glucose and HbA1c were significantly higher in T2DM patients than in normal controls (*P* < 0.05). There were no significant differences in age, BMI, HR, sex, DBP, TCH, TG, HDL and LDL between the normal controls and T2DM patients (*P* > 0.05).

The values of LVEF, E, E/A, eʹ and a′ in T2DM patients were significantly lower than those in normal controls (*P* < 0.05). There were no significant differences in RVd-base, RVd-mid, RVA-D, RVA-S, RVFAC, TAPSE, RVEDV, RVESV, RVEF, A, E/e′ and TVR between the normal controls and T2DM patients (*P* > 0.05).

### RV layer specific GLS and GLSr in normal controls and T2DM patients were shown in Table 2, Fig. 2

**Table2:** Layer specific RV GLS, GLSr between T2DM patients and normal subjects

Variable	Normal (59)	T2DM (52)	*t* value	*P* value
RVFW				
GLSEpi (%)	− 26.10 ± 4.02	− 23.39 ± 6.25	− 2.746	0.007
GLSMid (%)	− 28.68 ± 4.35	− 25.77 ± 6.43	− 2.827	0.006
GLSEndo (%)	− 32.09 ± 4.95	− 29.00 ± 6.87	− 2.740	0.007
GLSr-S (s^−1^)	− 1.82 ± 0.36	− 1.80 ± 0.38	− 0.216	0.830
GLSr-E (s^−1^)	2.03 ± 0.61	1.98 ± 0.58	0.433	0.666
GLSr-A (s^−1^)	1.28 ± 0.38	1.68 ± 0.65	− 3.916	< 0.001
RVFSW				
GLSEpi (%)	− 21.20 ± 3.46	− 19.47 ± 4.22	− 2.351	0.021
GLSMid (%)	− 23.92 ± 3.78	− 22.32 ± 4.54	− 1.998	0.048
GLSEndo (%)	− 27.61 ± 4.41	− 26.19 ± 5.19	− 1.541	0.126
GLSr-S (s^−1^)	− 1.54 ± 0.29	− 1.53 ± 0.28	− 0.302	0.763
GLSr-E (s^−1^)	1.77 ± 0.46	1.69 ± 0.44	0.923	0.358
GLSr-A (s^−1^)	1.11 ± 0.29	1.52 ± 0.46	− 5.424	< 0.001

*RVFW* right ventricle free wall, *RVFSW* right ventricle free-wall and septal wall, *GLSEpi* GLS of epimyocardial, *GLSMid* GLS of middle layer, *GLSEndo* GLS of endomyocardial, *GLSr-S* GLSr in systole, *GLSr-E* GLSr in early diastole, *GLSr-A* GLSr in late diastole

**Fig. 2:**
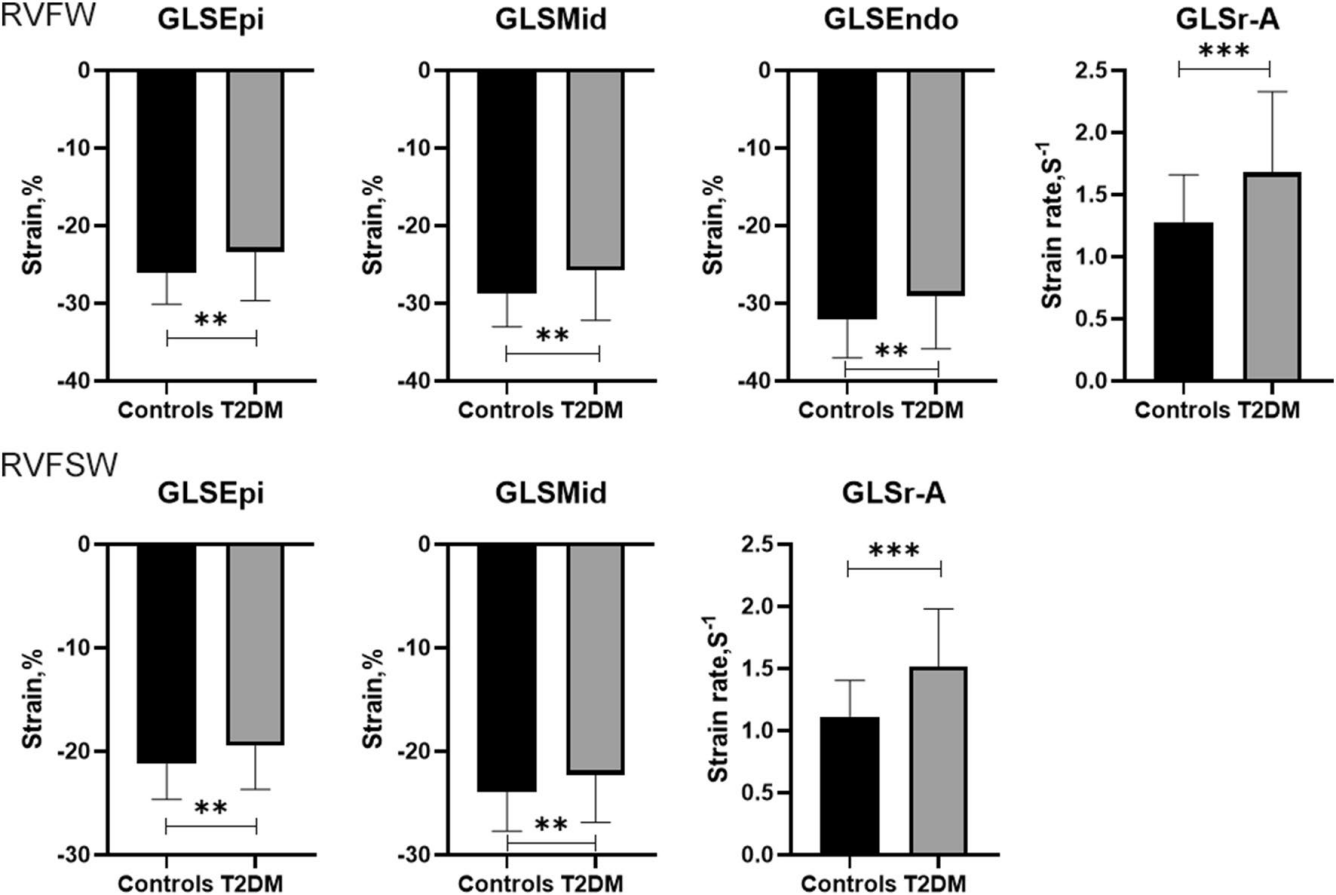
GLSEpi, GLSMid, GLSEndo, GLSr-A in RVFW and GLSEpi, GLSMid, GLSr-A in RVFSW between normal controls and T2DM patients (RVFW: right ventricle free wall, RVFSW: right ventricle free-wall and septal wall) (Independent Student′s t-test, normal: n = 59 and T2DM: n = 52, **means P < 0.01, ***means P < 0.001)

The absolute values of RV layer specific GLS (GLSEpi, GLSMid and GLSEndo) in RVFW in T2DM patients were significantly lower than normal controls (*P* < 0.01), while GLSr-A was significantly larger than normal controls (*P* < 0.001). There were no significant differences in GLSr-S and GLSr-E between normal controls and T2DM patients (*P* > 0.05).

The absolute values of RV layer specific GLS (GLSEpi and GLSMid) in RVFSW in T2DM patients were significantly lower than normal controls (*P* < 0.05), while GLSr-A was significantly larger than normal controls (*P* < 0.001). There were no significant differences in GLSEndo, GLSr-S and GLSr-E between normal controls and T2DM patients (*P* > 0.05).

### Correlation tests among biochemistry, echocardiography characters and layer specific RV GLS and GLSr-A values were shown in the Table 3 and 4, Fig. 3

**Table 3:** Correlation tests among fasting plasma glucose, HbA1c, RVFAC, RVEF, TAPSE and layer specific RV GLS in T2DM patients

Variable	RVFW						RVFSW			
	GLSEpi		GLSMid		GLSEndo		GLSEpi		GLSMid	
	*r* value	*P* value	*r* value	*P* value	*r* value	*P* value	*r* value	*P* value	*r* value	*P* value
Fasting plasma glucose	− 0.085	0.550	− 0.080	0.575	− 0.071	0.615	− 0.058	0.682	− 0.006	0.969
HbA1c	− 0.299	0.031	− 0.268	0.054	− 0.230	0.101	− 0.299	0.031	− 0.246	0.078
RVFAC	− 0.008	0.954	− 0.050	0.727	− 0.093	0.510	− 0.156	0.269	− 0.186	0.186
RVEF	− 0.080	0.575	− 0.088	0.536	− 0.097	0.494	− 0.107	0.450	− 0.107	0.451
TAPSE	0.059	0.679	0.007	0.963	− 0.059	0.676	0.089	0.531	0.010	0.946

**Table 4:** Correlation tests among fasting plasma glucose, HbA1c, E, A, E/A, eʹ, aʹ, E/eʹ and RV GLSr-A in T2DM patients

Variable	RVFW		RVFSW	
	GLSr-A		GLSr-A	
	*r* value	*P* value	*r* value	*P* value
Fasting plasma glucose	0.145	0.306	0.098	0.489
HbA1c	0.179	0.204	0.157	0.266
E	0.080	0.575	0.087	0.539
A	0.203	0.149	0.242	0.085
E/A	− 0.109	0.440	− 0.146	0.301
eʹ	0.007	0.962	0.076	0.594
aʹ	0.051	0.721	0.100	0.483
E/eʹ	− 0.037	0.793	− 0.093	0.511

**Fig. 3:**
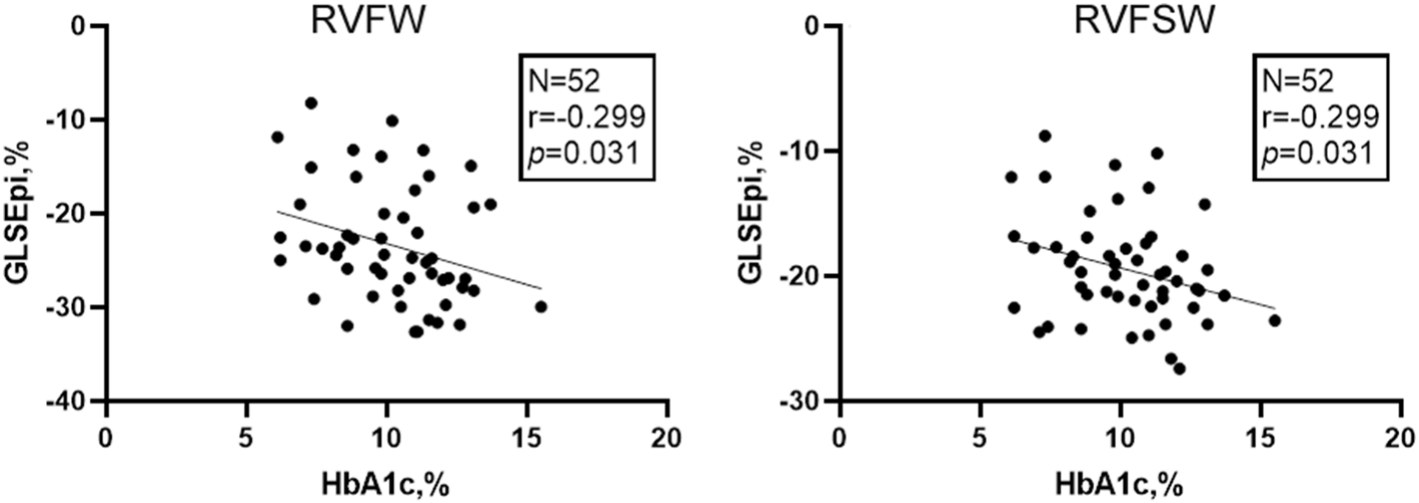
Correlation tests showed HbA1c was negatively correlated with GLSEpi in RVFW and RVFSW in T2DM patients (RVFW: right ventricle free wall, RVFSW: right ventricle free-wall and septal wall) (Pearson correlation tests, T2DM: n = 52)

HbA1c were negatived correlated with GLSEpi in RVFW and RVFSW (*P* < 0.05) in T2DM patients. There were no significant correlations among the other values (*P* > 0.05).

### ROC analysis to confirm the diagnostic values of RV systolic and diastole function by GLS and GLSr in RVFW and RVFSW were shown in Table 5, Fig. 4

**Table 5:** Receiver operating characteristic curve analysis for the detection RV systolic and diastole dysfunctions in T2DM patients

Variable	RVFW				RVFSW		
	GLSEpi	GLSMid	GLSEndo	GLSr-A	GLSEpi	GLSMid	GLSr-A
AUC (SE)	0.581	0.594	0.602	0.701	0.602	0.590	0.773*
AUC (95% CI)	0.480–0.677	0.493–0.689	0.502–0.697	0.607–0.784	0.505–0.694	0.493–0.683	0.684–0.847
Cut-off value	− 25.70	− 27.72	− 31.73	1.56	− 21.83	− 22.38	1.16
Sensitivity	55.77	53.85	63.46	59.62	73.08	51.92	78.85
Specificity	61.54	65.38	55.77	79.66	45.76	67.80	64.41
Youden index	0.1731	0.1923	0.1923	0.3928	0.1884	0.1972	0.4325

^*^means *P* < 0.05 when GLSr-A in RVFSW compared with other values

**Fig. 4:**
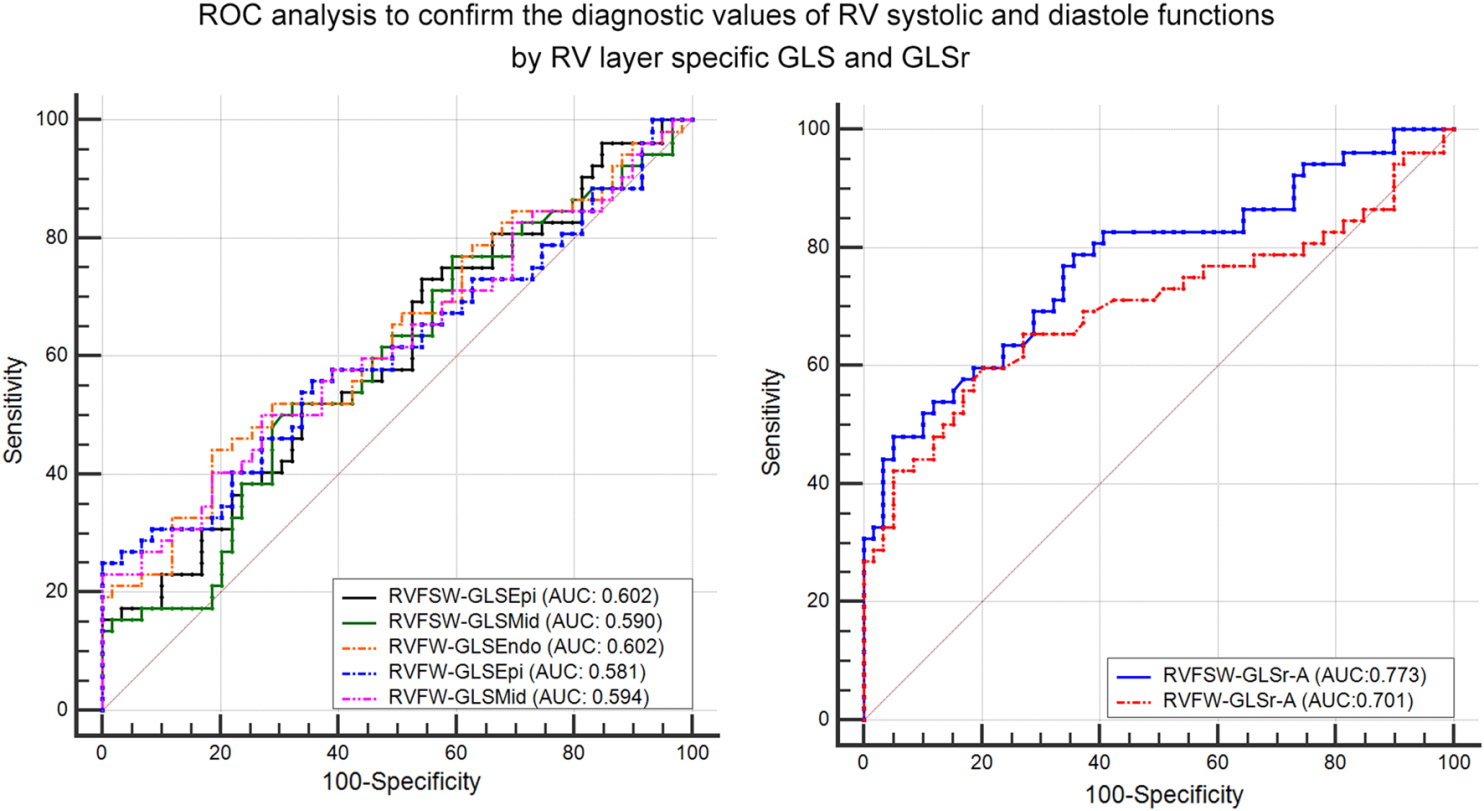
ROC analysis for detecting the accuracy of RV systolic and diastole dysfunctions in T2DM patients (ROC analysis, normal: n = 59, and T2DM: n = 52)

In RVFW: the area under the curve (AUC) of GLSEpi was 0.581, (95% CI) was 0.480–0.677, the cut-off value was − 25.7%, with a sensitivity of 55.77% and specificity of 61.54%. The AUC of GLSMid was 0.594, (95% CI) was 0.493–0.689, the cut-off value was − 27.72%, with a sensitivity of 53.85% and specificity of 65.38%. The AUC of GLSEndo was 0.602, (95% CI) was 0.502–0.697, the cut-off value was − 31.73%, with a sensitivity of 63.46% and specificity of 55.77%. The AUC of GLSr-A was 0.701, (95% CI) was 0.607–0.784, the cut-off value was 1.56 s^−1^, with a sensitivity of 59.62% and specificity of 79.66%.

In RVFSW: The AUC of GLSEpi was 0.602, (95% CI) was 0.505–0.694, the cut-off value was − 21.83%, with a sensitivity of 73.08% and specificity of 45.76%. The AUC of GLSMid was 0.590, (95% CI) was 0.493–0.683, the cut-off value was − 22.38%, with a sensitivity of 51.92% and specificity of 67.80%. The AUC of GLSr-A was 0.773, (95% CI) was 0.684–0.847, the cut-off value was 1.16 s^−1^, with a sensitivity of 78.85% and specificity of 64.41%.

There were significant differences between GLSr-A in RVFSW and other above mentioned diagnostic values (all *P*<0.05).

## Discussion

The study indicated that the subclinical impairment both in RV systolic and diastole functions were detected by layer specific GLS and GLSr in asymptomatic T2DM patients with poor glycemic control.

RV function is difficult to evaluate in echocardiography because of its complex geometry. Addetia K, et al. [13] used RV systolic parameters, such as basal and mid-RV dimensions, length, TAPSE, tissue Doppler S′ velocity, and RV longitudinal strain in normal subjects, and found that RV strain was believed to better represent RV systolic function than longitudinal measurements. Previous studies mainly focused on some diseases may damage RV functions directly, such as pulmonary arteries hypertension [14, 15], atrial septal defect [16], tetralogy of Fallot [17, 18], severe tricuspid regurgitation [19], and so on. Although there were some studies on RV function, the information on RV systolic and diastole functions in T2DM patients using RV layer specific GLS and GLSr was still limited.

Todo S, et al. [3] used RV free‑wall strain to investigate RV systolic dysfunction and its association with LV longitudinal myocardial dysfunction in T2DM patients with normal LVEF, and observed that RV subclinical systolic dysfunction was associated with LV longitudinal myocardial dysfunction. Vittos O, et al. [1] used inflammatory biomarkers, adiponectin and RV strain and strain rate properties in T2DM patients, and found that a low-grade inflammatory status correlated with RV systolic dysfunction. From the research, partial results were in accordance with the previous studies, and we found that RV dysfunctions in T2DM patients. However, we had also grown the potential information of RV impaired systolic and diastole functions. Layer specific GLS in RVFW and RVFSW concluded that RV impaired systolic function in a more subtle way. GLSr-A in RVFW and RVFSW concluded that RV impaired diastolic function. As we know, LV involvement in T2DM patients has been demonstrated by large studies. In T2DM, hypoxia of cardiomyocytes and ischaemia results in myocardial hypertrophy, perivascular and fibrosis, LV stiffness, and systolic and diastole dysfunctions in T2DM [20]. However, LV involvement in diabetic cardiomyopathy is systemic changes and therefore could hamper RV functions, also contain the systolic and diastole functions [3]. The muscle layer of the RV myocardial wall is primarily composed of longitudinal fibers [10], and from the research, impaired longitudinal layer specific RV strain and strain rate could explain the predominance of longitudinal RV systolic and diastole functions changes.

ROC analysis showed that layer specific GLS and GLSr-A had the accurate diagnostic efficacy in T2DM patients, and GLSr-A in RVFSW get the best diagnostic value in RV diastolic function in T2DM patients. The results may indicate layer specific GLS and GLSr-A can evaluate the RV systolic and diastole dysfunctions in T2DM patients accurately.

In T2DM patients, HbA1c were negatived correlated with GLSEpi in RVFW and RVFSW in T2DM patients. However, the correlations were not strong (only 0.299) and they could not accurately predict the RV myocardial systolic and diastolic dysfunctions in T2DM patients with normal LV and RV systolic functions.

## Conclusion

From the research, we found that layer specific strain and strain rate could detect the RV myocardial dysfunctions and confirmed that the impaired RV systolic and diastole functions in T2DM patients with poor glycemic control. GLSr-A in RVFSW had the best diagnostic value in evaluating RV diastolic function in T2DM patients.

## Data Availability

The datasets used and analyzed during the current study are available from the corresponding author on reasonable request.

## Abbreviations

LV: Left ventricle
RV: Right ventricle
STI: Speckle-tracking imaging
T2DM: Type 2 diabetes mellitus
GLS: Global longitudinal strain
GLSr: Global longitudinal strain rate
BMI: Body mass index
SBP: Systolic blood pressure
DBP: Diastolic blood pressure
HbA1c: Glycated haemoglobin
TCH: Total cholesterol
TG: Triglyceride
HDL: High density lipoprotein
LDL: Low density lipoprotein
RVd-base: Right ventricle basal diameter
RVd-mid: RV middle diameter
RVA-D: RV areas in the diastole period
RVA-S: RV areas in the systole period
RVFAC: RV fractional area change
RVEDV: RV end diastole volume
RVESV: RV end systole volume
RVEF: Right ventricular ejection fraction
LVEF: Left ventricular ejection fraction
E: Peak velocity during early diastole of tricuspid valve
A: Peak velocity during late diastole of tricuspid valve
e′: Peak early diastolic velocities of the anterior tricuspid annulus using TDI
a′: Peak late diastolic velocities of the anterior tricuspid annulus using TDI
TRV: Tricuspid regurgitation velocity
